# Spotted Temporal Lobe Necrosis following Concurrent Chemoradiation Therapy Using Image-Guided Radiotherapy for Nasopharyngeal Carcinoma

**DOI:** 10.1155/2022/5877106

**Published:** 2022-09-27

**Authors:** Yu-Wei Chiang, Li-Jen Liao, Chia-Yun Wu, Wu-Chia Lo, Pei-Wei Shueng, Chen-Xiong Hsu, Deng-Yu Guo, Pei-Yu Hou, Pei-Ying Hsieh, Chen-Hsi Hsieh

**Affiliations:** ^1^Faculty of Medicine, School of Medicine, National Yang Ming Chiao Tung University, Taipei 112, Taiwan; ^2^Otolaryngology, Far Eastern Memorial Hospital, New Taipei City 220, Taiwan; ^3^Head and Neck Cancer Surveillance and Research Group, Far Eastern Memorial Hospital, New Taipei City 220, Taiwan; ^4^Department of Electrical Engineering, Yuan Ze University, Taoyuan 320, Taiwan; ^5^Department of Oncology and Hematology, Far Eastern Memorial Hospital, New Taipei City 220, Taiwan; ^6^Graduate Institute of Medicine, Yuan Ze University, Taoyuan 320, Taiwan; ^7^Division of Radiation Oncology, Far Eastern Memorial Hospital, New Taipei City 220, Taiwan; ^8^Department of Biomedical Imaging and Radiological Sciences, National Yang Ming Chiao Tung University, Taipei 112, Taiwan; ^9^Institute of Traditional Medicine, School of Medicine, National Yang Ming Chiao Tung University, Taipei 112, Taiwan

## Abstract

**Background:**

To explore spotted temporal lobe necrosis (TLN) and changes in brain magnetic resonance imaging (MRI) after image-guided radiotherapy (IGRT) in a patient with nasopharyngeal carcinoma (NPC). Case presentation: a 57-year-old male was diagnosed with stage III NPC, cT1N2M0, in 2017. He underwent concurrent chemoradiation therapy (CCRT) with cisplatin (30 mg/m^2^) and 5- fluorouracil (5-FU, 500 mg/m^2^) plus IGRT with 70 Gy in 35 fractions for 7 weeks. The following MRI showed a complete response in the NPC. However, the patient suffered from fainting periodically when standing up approximately 3 years after CCRT. Neck sonography showed mild atherosclerosis (< 15%) of bilateral carotid bifurcations and bilateral small-diameter vertebral arteries, with reduced flow volume. The following MRI showed a 9 mm × 7 mm enhancing lesion in the right temporal lobe without locoregional recurrence, and TLN was diagnosed. The lesion was near the watershed area between the anterior temporal and temporo-occipital arteries. The volume of the necrotic lesion was 0.51 c.c., and the mean dose and Dmax of the lesion were 64.4 Gy and 73.7 Gy, respectively. Additionally, the mean dose, V45, D1 c.c. (dose to 1 ml of the temporal lobe volume), D0.5 c.c. and Dmax of the right and left temporal lobes were 11.1 Gy and 11.4 Gy, 8.5 c.c. and 6.7 c.c., 70.1 Gy and 67.1 Gy, 72.0 Gy and 68.8 Gy, and 74.2 Gy and 72.1 Gy, respectively.

**Conclusion:**

Spotted TLN in patients with NPC treated by IGRT may be difficult to diagnose due to a lack of clinical symptoms and radiological signs. Endothelial damage may occur in carotid and vertebral arteries within the irradiated area, affecting the small branches supplying the temporal lobe and inducing spotted TLN. Future research on the relationship between vessels and RT or CCRT and the development of TLN is warranted.

## 1. Introduction

Temporal lobe necrosis (TLN) is one of the potential long-term complications of radiotherapy (RT) or concurrent chemoradiotherapy (CCRT) in patients with nasopharyngeal carcinoma (NPC) [1]. The incidence of TLN is approximately 3% to 35% in 3.5 to 10 years after treatment with conventional 2-dimensional (2D) or 3-dimensional (3D) conformal RT for T1-4 NPC [2, 3]. Most NPC patients with TLN are asymptomatic; however, other patients may experience epilepsy, dysphasia, seizures, cognitive decline, changes in consciousness, memory impairment, dizziness, and headaches [4].

Intensity-modulated radiotherapy (IMRT) is a modern technique of RT for NPC. Volume-modulated arc therapy (VMAT) and helical tomotherapy (HT), image-guided arc radiotherapy techniques, are receiving additional attention compared with conventional IMRT for patients with NPC [5]. HT delivers highly conformal dose distributions for head and neck cancer with an impressive ability to spare critical organs [6, 7]. Compared to VMAT and IMRT in patients with NPC, HT presents a sharp dose gradient associated with optimal conformity, homogeneity, and better performance in sparing surrounding organs at risk (OARs) [5].

The dwindling 5-year incidence of TLN in NPC patients treated with IMRT is significantly higher than that in NPC patients treated with conventional RT, by 20% [8]. However, TLN is still a significant complication due to the clinical and radiological difficulties in distinguishing it from malignancy and the possibility of incapacitating changes even using modern radiation techniques. Herein, we present the case of a patient with NPC who underwent CCRT with an image-guided technique and developed spotted TLN.

## 2. Case Report

A 57-year-old male without diabetes mellitus or hypertension visited our hospital for right ear tinnitus, hearing loss for two weeks, and periodic hemoptysis for one month in November 2017. A mass with bleeding on contact was found in the nasopharyngeal area on fibroscopy. The mass at the nasopharynx was diagnosed as undifferentiated nonkeratinizing carcinoma by pathology. Magnetic resonance imaging (MRI) showed a 2.4 *∗* 1.7 *∗* 2 cm^3^ mucosal mass in the bilateral nasopharynx with clustered, enlarged lymph nodes at level IIb on the left and several small retropharyngeal lymph nodes bilaterally at levels Ib and II. Additionally, positron emission tomography-computed tomography (PET-CT) showed hypermetabolic nodules in the neck bilaterally at level III and on the left at levels IIA and V, and stage III NPC, cT1N2M0 were diagnosed (American Joint Committee on Cancer, 7th Edition). The patient subsequently underwent CCRT comprising weekly cisplatin (30 mg/m^2^) and 5-FU (500 mg/m^2^) as well as HT with 70 Gy in 35 fractions for 7 weeks. After CCRT was completed, MRI suggested a complete response in the NPC. Regular follow-up MRI and fibroscopy examinations were arranged and showed no evidence of locoregional recurrence.

**Figure 1:**
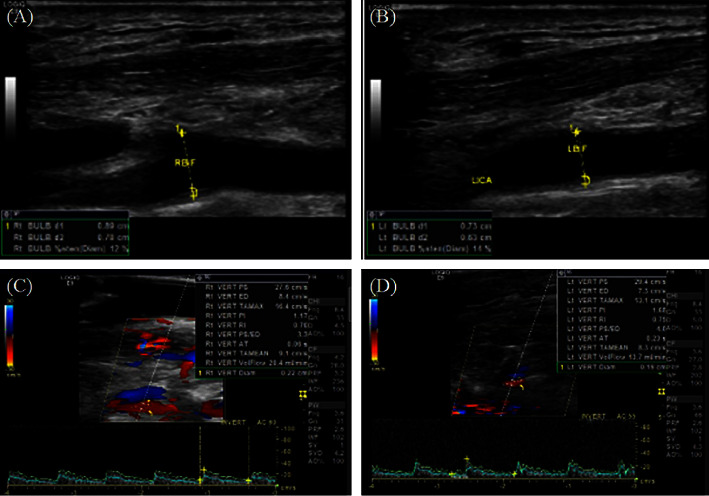
Neck ultrasound for carotid artery study. The data showed mild atherosclerosis (< 15%) of bilateral carotid bifurcations and bilateral small-diameter vertebral arteries, with reduced flow volume (34 ml/min). (a) Right carotid bifurcation. (b) Left carotid bifurcation. (c) Right vertebral artery. (d) Left vertebral artery.

**Figure 2:**
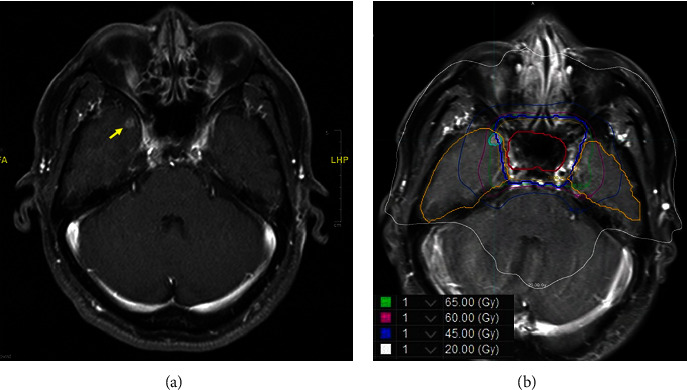
Magnetic resonance imaging (MRI) showed (a) a small enhancing focal lesion in the right temporal pole with a size of 9 mm × 7 mm, and brain radiation necrosis was suspected (arrow indicates the lesion with rim enhancement). (b) Superimposition of cumulative radiation dose distribution on transverse postcontrast MRI showing the relation between the radiation dose and lesion with rim enhancement (indicated with aqua).

However, the patient began to suffer from fainting periodically when standing up in March 2021. Neck sonography showed mild atherosclerosis (<15%) of bilateral carotid bifurcations and bilateral small-diameter vertebral arteries, with reduced flow volume (Figures 1(a)–1(d)). Five months later, a neck MRI showed a small enhancing focal lesion in the right temporal pole with a size of 9 mm × 7 mm, and brain metastasis was suspected (Figure 2(a)). Regular MRI every 3 months until April 2022 showed stable spotted rim enhancement on postcontrast T1 imaging, mild hyperenhancement on T2 imaging, and no diffusion restriction on diffusion-weighted imaging, and radiation necrosis was diagnosed. The volume of the necrotic lesion was 0.51 c.c., and the mean dose and Dmax of the lesion were 64.4 Gy and 73.7 Gy, respectively. Additionally, the mean dose, V45, D1 c.c. (dose to 1 ml of the temporal lobe volume), D0.5 c.c. and Dmax of the right and left temporal lobes were 11.1 Gy and 11.4 Gy, 8.5 c.c. and 6.7 c.c., 70.1 Gy and 67.1 Gy, 72.0 Gy and 68.8 Gy, and 74.2 Gy and 72.1 Gy, respectively (Figure 2(b)). Data were collected retrospectively with approval from the Institutional Review Board of the Far Eastern Memorial Hospital (FEMH-IRB-111147-C), and the requirement for informed consent was waived.

## 3. Discussion

Radiation necrosis consists of necrotic changes in tissue following RT and typically develops one to four years after RT [8, 9]. The risk of TLN is highly dependent on the presence of high-dose “hot spots” in the temporal lobe. Huang et al. [10] suggested that a D1 c.c. of 71.1 Gy can be used to predict TLN, while Sun et al. reported a mean D0.5 c.c. of 73.5 Gy ± 7.3 Gy for symptomatic TLN [11]. Additionally, a V45 less than 15.1 cm^3^ is considered a relatively safe constraint for preventing TLN lesions [12]. Moreover, the average maximum dose deposited in TLN areas by IMRT has been reported to be 76.7 Gy [13].

The patient in the current case experienced TLN 3 years after CCRT was completed. Interestingly, one study found a significantly shorter onset time in those treated with IMRT than in those treated with conventional RT (36.9 months vs. 49.8 months) [8]. In the present case, the volume of the necrotic lesion was 0.51 c.c., and the Dmax of the lesion was 73.7 Gy. Additionally, the V45, D1 c.c. and D0.5 c.c. of the right and left temporal lobes were 8.5 c.c. and 6.7 c.c., 70.1 Gy and 67.1 Gy, and 72.0 Gy and 68.8 Gy, respectively. While these data are in line with the suggested parameters mentioned above, the patient still developed TLN; additionally, the lesion presented a spotted appearance, which is different from that of other large necrotic areas in the brain reported in the literature [11].

The vascular injury theory and the glial injury theory have been proposed to explain cerebral radiation necrosis [14]. According to the glial injury theory, irradiation directly injures the brain parenchyma which creates demyelination in the white matter [15]. However, this hypothesis is an argument because sublethal radiation doses cause the number of glial cells to decrease but may not result in histological necrosis changes [16]. The quantitative analysis of normal tissue effects in the Clinic (QUANTEC) study reported a risk of 5% for radiation necrosis of the brain at 5 years after normally fractionated RT with 72 Gy [17]. In our case, the site of necrosis was located in the 60 Gy dose-line area. However, other brain tissue in the 60 Gy dose-line area did not develop necrosis. Therefore, the glial injury theory does not seem to completely explain such spotted radiation necrosis.

The other hypothesis is the vascular injury theory. Based on the histopathological examination of human brain radiation necrosis surgical specimens, Yoritsune et al. [18] suggested that blood vessel damage caused by radiotherapy could result in the change of radiation necrosis. When the vessels are exposed to radiation, endothelial progenitor cells undergo apoptosis and can lead to atheroma formation [19]. Additionally, radiation also stimulates the activation of chemokines, cytokines, proteases, growth factors, and regulators, as well as adhesion molecules, to cause inflammatory reactions [20]. These chain reactions culminate in inflammation of the vessels and atherosclerotic plaque formation and eventually lead to fibrinoid necrosis of the vessel wall, ischemia, edema, and cell death [21], which can contribute to vessel stenosis, coronary artery disease, and stroke.

**Figure 3:**
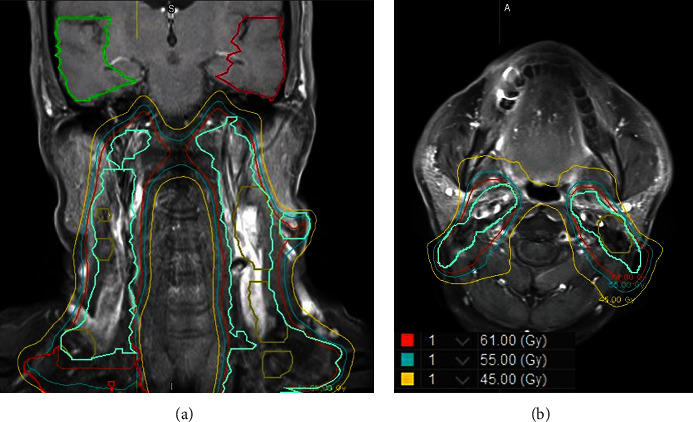
The patient's carotid artery system was within the irradiation area. (a) Coronal view. (b) Transverse view.

The arterial supply to the temporal lobe has two main sources, including the internal carotid system and the vertebrobasilar system. The internal carotid system contains the anterior choroidal artery and middle cerebral artery, which then branches into the temporopolar artery and anterior temporal artery. On the other hand, the vertebrobasilar system through the temporo-occipital artery supplies the inferior surface of the temporal lobe. In this case, the patient experienced TLN more than 3 years after CCRT was completed. Additionally, the lesion in the case was near the watershed area between the anterior temporal and temporo-occipital arteries. Furthermore, the patient's carotid artery system was within the irradiation area (Figures 3(a) and 3(b)), and carotid sonography showed atherosclerosis of bilateral carotid bifurcations and bilateral small-diameter vertebral arteries, with reduced flow volume.

RT itself can also cause vascular injury. Dimitrievich et al. [22] reported that the radiosensitivity of capillaries was significantly greater than that of larger vessels. Zou et al. [23] reported that high-grade occlusive radiation vasculopathy lesions diffusely involved the common carotid artery and internal carotid artery and were associated with complete occlusion in one or both carotid arteries and vertebral artery steno-occlusion. Moreover, the common/internal carotid arteries are the most vulnerable arteries after RT [24]. The radiation dose [17, 25] and postradiation interval [26, 27] are both risk factors for carotid stenosis. Ye et al. [28] reported that plaques were more common in patients after RT, especially in those with TLN.

Importantly, CCRT also increases the risk of developing radiation necrosis [29]. Both 5-FU and cisplatin have been linked to an increased risk of stroke [30]. Cisplatin-based CCRT causes systemic vascular inflammation with endothelial dysfunction that may contribute to atherothrombotic events [31, 32]. Likewise, 5-FU produces similar vascular toxicity by directly damaging endothelial cells [33]. Furthermore, Sneed et al. [34] reported that CCRT with capecitabine plus 5-FU resulted in a higher incidence of radiation necrosis. As aforementioned, spotted TLN may show dissimilar manifestations of vascular or arterial injury, and the potential relation of this damage to RT or CCRT with cisplatin plus 5-FU cannot be excluded.

The current management strategy for radiation necrosis depends on whether the patient is symptomatic. For asymptomatic patients, conservative treatment is suggested. For symptomatic patients, steroids are normally the first-line treatment for decreasing cerebral edema and inflammation [35]. For steroid-refractory patients, bevacizumab is usually suggested and can result in a nearly twofold radiographic response and significant clinical improvement compared with corticosteroids [36]. Pasquier et al [37] reported limited promising retrospective reports of hyperbaric oxygen (HBO) therapy for brain radiation necrosis; however, no relevant prospective data are available to date. Similarly, no prospective trials of surgical resection for brain radiation necrosis have been reported. Telera et al. [38] reported the partial or complete tapering of corticosteroids as well as symptom improvement in the majority of patients with brain radiation necrosis after surgical resection. The first multicenter study of laser-induced thermal therapy (LITT) was published by Chaunzwa et al. [39]. The data showed that a total of 73.3% of patients stopped steroids and that 48% saw an improvement in their preoperative symptoms after LITT.

## 4. Conclusion

Spotted TLN in patients with NPC treated by IGRT is not easy to diagnose due to clinical and radiological difficulties in distinguishing it from other conditions. Various parameters of the RT strategy, including the total dose, dosimetry parameters, radiation fields, and the presence of vessels in the radiation fields, contribute to TLN. However, the presence of carotid and vertebral arteries within the irradiated area may increase the risk of TLN due to vascular endothelial injury. Future research on the relationship between vascular endothelial injury and RT and the development of TLN is warranted.

## Data Availability

The data used to support the findings of this study are included within the article.
